# Supplementary material for: Unplanned Out-of-Hospital Birth—Short and Long-Term Consequences for the Offspring
Source: J Clin Med. 2020 Jan 25;9(2):339. doi: 10.3390/jcm9020339 (PMC7073687; doi:10.3390/jcm9020339)
Supplement: Supplementary file 1 [file jcm-09-00339-s001.pdf]

Supplementary Tables – ICD-9 Codes

Neurological morbidity

GROUP	DIAG. CODE	DIAGNOSIS DESCRIPTION
**AUTISM**	2990	AUTISTIC DISORDER
	2990	INFANTILE AUTISM
	2998	OTHER SPECIFIED PERVASIVE DEVELOPMENTAL DISORDERS
	29900	AUTISTIC DISORDER, CURRENT OR ACTIVE STATE
	29901	AUTISTIC DISORDER, RESIDUAL STATE
	29910	CHILDHOOD DISINTEGRATIVE DISORDER, CURRENT OR ACTIVE STATE
	29981	OTHER SPECIFIED PERVASIVE DEVELOPMENTAL DISORDERS, RESIDULA STATE
	29990	UNSPECIF.PERVASIVE DEVELOPMENTAL DISORDER, CURRENT OR ACTIVE STATE
**EATING DISORDERS**	3071	ANOREXIA NERVOSA
	3075	OTHER AND UNSPECIFIED DISORDERS OF EATING
	30750	EATING DISORDER, UNSPECIFIED
	30751	BULIMIA NERVOSA
	30753	RUMINATION DISORDER
	30759	OTHER DISORDERS OF EATING
	V691	INAPPROPRIATE DIET & EATING HABITS
**SLEEPING DISORDERS**	7805	SLEEP DISTURBANCES
	30746	SLEEP AROUSAL DISORDER
	30746	SOMNAMBULISM OR NIGHT TERRORS
	30747	OTHER DYSFUNCTIONS OF SLEEP STAGES OR AROUSAL FROM SLEEP
	32730	CIRCADIAN RHYTHM SLEEP DISORDER, UNSPECIFIED
	32732	CIRCADIAN RHYTHM SLEEP DISORDER, ADVANCED SLEEP PHASE TYPE
	34700	NARCOLEPSY WITHOUT CATAPLEXY
	34701	NARCOLEPSY WITH CATAPLEXY
	78050	UNSPECIFIED SLEEP DISTURBANCE
	78052	INSOMNIA, UNSPECIFIED
	78052	OTHER INSOMNIA
	78054	HYPERSOMNIA, UNSPECIFIED
	78056	DYSFUNCTIONS ASSOCIATED WITH SLEEP STAGES OR AROUSAL FROM SLEEP
	78059	OTHER SLEEP DISTURBANCES
	V694	LACK OF ADEQUATE SLEEP
**MOVEMENT DISORDERS**	3073	STEREOTYPIC MOVEMENT DISORDER
	3331	ESSENTIAL AND OTHER SPECIFIED FORMS OF TREMOR
	3332	MYOCLONUS
	3335	OTHER CHOREAS
	3336	GENETIC TORSION DYSTONIA
	3336	IDIOPATHIC TORSION DYSTONIA
	3343	OTHER CEREBELLAR ATAXIA
	3450	GENERALIZED NONCONVULSIVE EPILEPSY
	3452	PETIT MAL STATUS, EPILEPTIC

	3453	GRAND MAL STATUS, EPILEPTIC
	3455	PARTIAL EPILEPSY, WITHOUT IMPAIRMENT OF CONSCIOUSNESS
	3456	INFANTILE SPASMS
	3459	EPILEPSY, UNSPECIFIED
	7810	ABNORMAL INVOLUNTARY MOVEMENTS
	7812	ABNORMALITY OF GAIT
	7813	LACK OF COORDINATION
	33390	UNSP.EXTRAPYRAMIDAL DISEASE + ABNORMAL MOVEMENT DISORDER
	33399	OTHER EXTRAPYRAMIDAL DISEASES AND ABNORMAL MOVEMENT DISORDERS
	34500	GENERALIZED NONCONVULSIVE EPILEPSY WITHOUT INTRACTABLE EPILEPSY
	34501	GENERALIZED NONCONVULSIVE EPILEPSY WITH INTRACTABLE EPILEPSY
	34510	GENERALIZED CONVULSIVE EPILEPSY WITHOUT INTRACTABLE EPILEPSY
	34511	GENERALIZED CONVULSIVE EPILEPSY WITH INTRACTABLE EPILEPSY
	34540	PARTIAL EPILEPSY+IMPAIRMENT OF CONSCIOUSNESS WITHOUT INTRACTABLE EPILEPSY
	34550	PARTIAL EPILEPSY WITHOUT IMPAIRMENT OF CONSCIOUSNESS WITHOUT INTR ACTABEL EPILEPSY
	34560	INFANTILE SPASMS WITHOUT INTRACTABLE EPILEPSY
	34590	EPILEPSY, NUSP. WITHOUT INTRACTABEL EPILEPSY
	34590	EPILEPSY, UNSP. WITHOUT INTRACTABLE EPILEPSY
	34591	EPILEPSY UNSP. WITH INTRACTABLE EPILEPSY
	78031	FEBRILE CONVULSIONS
	78031	FEBRILE CONVULSIONS (SIMPLE), UNSPECIFIED
	78032	COMPLEX FEBRILE CONVULSIONS
	78039	OTHER CONVULSIONS
	78099	OTHER GENERAL SYMPTOMS
**CEREBRAL PALSY**	3341	HEREDITARY SPASTIC PARAPLEGIA
	3421	SPASTIC HEMIPLEGIA
	3429	HEMIPLEGIA, UNSPECIFIED
	3430	CONGENITAL DIPLEGIA
	3431	CONGENITAL HEMIPLEGIA
	3432	CONGENITAL QUADRIPLEGIA
	3439	INFANTILE CEREBRAL PALSY, UNSPECIFIED
	3441	PARAPLEGIA
	3442	DIPLEGIA OF UPPER LIMBS
	3449	PARALYSIS, UNSPECIFIED
	3481	ANOXIC BRAIN DAMAGE
	3526	MULTIPLE CRANIAL NERVE PALSIES
	7814	TRANSIENT PARALYSIS OF LIMB
	34210	SPASTIC HEMIPLEGIA AFFECTING UNSP. SIDE
	34290	HEMIPLEGIA, UNSP., AFFECTING UNSP. SIDE
	34291	HEMIPLEGIA, UNSP., AFFECTING DOMINANT SIDE
	34292	HEMIPLEGIA, UNSP., AFFECTING NONDOMINANT SIDE
	34400	QUADRIPLEGIA, UNSPECIFIED
	34430	MONOPLEGIA OF LOWER LIMB, AFFECTING UNSP. SIDE
	34440	MONOPLEGIA OF UPPER LIMB, AFFECTING UNSP. SIDE

	34489	OTHER SPECIFIED PARALYTIC SYNDROME
	43811	APHASIA
	43820	HEMIPLEGIA AFFECTING UNSP. SIDE
**PSYCHIATRIC DISORDERS**	309	ADJUSTMENT REACTION
	311	DEPRESSIVE DISORDER, NOT ELSEWHERE CLASSIFIED
	316	PSYCHIC FACTORS ASSOCIATED WITH DISEASES CLASSIFIED ELSEWHERE
	2930	ACUTE DELIRIUM
	2930	DELIRIUM DUE TO CONDITIONS CLASSIFIED ELSEWHERE
	2940	AMNESTIC DISORDER IN CONDITIONS CLASSIFIED ELSEWHERE
	2949	UNSPECIFIED PERSISTENT MENTAL DISORDERS DUE TO COND.CLASS.ELSEWH.
	2971	DELUSIONAL DISORDER
	2979	UNSPECIFIED PARANOID STATE
	2981	EXCITATIVE TYPE PSYCHOSIS
	2983	ACUTE PARANOID REACTION
	2989	UNSPECIFIED PSYCHOSIS
	3003	OBSESSIVE-COMPULSIVE DISORDERS
	3004	DYSTHYMIC DISORDER
	3004	NEUROTIC DEPRESSION
	3009	UNSPECIFIED NONPSYCHOTIC MENTAL DISORDER
	3019	UNSPECIFIED PERSONALITY DISORDER
	3026	DISORDERS OF PSYCHOSEXUAL IDENTITY
	3051	TOBACCO USE DISORDER (TOBACCO DEPENDENCE)
	3061	RESPIRATORY MALFUNCTION ARISING FROM MENTAL FACTORS
	3062	CARDIOVASCULAR MALFUNCTION ARISING FROM MENTAL FACTORS
	3068	OTHER SPECIFIED PSYCHOPHYSIOLOGICAL MALFUNCTION
	3069	UNSPECIFIED PSYCHOPHYSIOLOGICAL MALFUNCTION
	3070	ADULT ONSET FLUENCY DISORDER
	3070	STAMMERING AND STUTTERING
	3070	STUTTERING
	3080	PREDOMINANT DISTURBANCE OF EMOTIONS
	3089	UNSPECIFIED ACUTE REACTION TO STRESS
	3090	ADJUSTMENT DISORDER WITH DEPRESSED MOOD
	3094	ADJUSTMENT DISOR.WITH MIXED DISTURB.OF EMOTIONS AND CONDUCT
	3099	UNSPECIFIED ADJUSTMENT REACTION
	3129	UNSPECIFIED DISTURBANCE OF CONDUCT
	3139	UNSPECIFIED EMOTIONAL DISTURBANCE OF CHILDHOOD OR ADOLESCENCE
	7801	HALLUCINATIONS
	7803	CONVULSIONS
	7992	NERVOUSNESS
	7993	DEBILITY, UNSPECIFIED
	29384	ANXIETY DISORDER IN CONDITIONS CLASSIFIED ELSEWHERE
	29530	PARANOID TYPE SCHIZOPHRENIA, UNSPECIFIED STATE
	29570	SCHIZOAFFECTIVE DISORDER SCHIZOPHRENIA, UNSPECIFIED STATE
	29580	OTHER SPECIFIED TYPES OF SCHIZOPHRENIA, UNSPECIFIED STATE
	29590	UNSPECIFIED TYPE SCHIZOPHRENIA, UNSPECIFIED STATE

	29600	BIPOLAR I DISORDER, SINGLE MANIC EPISODE, UNSPECIFIED DEGREE
	29620	MAJOR DEPRESSIVE AFFECTIVE DISORDER, SINGLE EPISODE, UNSP.DEGREE
	29680	BIPOLAR DISORDER, UNSPECIFIED
	29690	UNSPECIFIED EPISODIC MOOD DISORDER
	29699	OTHER SPECIFIED AFFECTIVE PSYCHOSES
	30000	ANXIETY STATE, UNSPECIFIED
	30001	PANIC DISORDER WITHOUT AGORAPHOBIA
	30009	OTHER ANXIETY STATES
	30010	HYSTERIA, UNSPECIFIED
	30011	CONVERSION DISORDER
	30029	OTHER ISOLATED OR SIMPLE PHOBIAS
	30183	BORDERLINE PERSONALITY
	30183	BORDERLINE PERSONALITY DISORDER
	30302	AC. ALCOHOLIC INTOXIC.IN ALCOHOLISM,EPISODIC DRINKING BEHAVIOR
	30400	OPIOID TYPE DEPENDENCE, UNSPECIFIED USE
	30430	CANNABIS DEPENDENCE, UNSPECIFIED USE
	30432	CANNABIS DEPENDENCE, EPISODIC USE
	30500	ALCOHOL ABUSE, UNSPECIFIED DRINKING BEHAVIOR
	30501	ALCOHOL ABUSE, CONTINUOUS DRINKING BEHAVIOR
	30502	ALCOHOL ABUSE, EPISODIC DRINKING BEHAVIOR
	30591	OTHER, MIXED, OR UNSPECIFIED DRUG ABUSE, CONTINUOUS USE
	30720	TIC DISORDER, UNSPECIFIED
	30722	CHRONIC MOTOR OR VOCAL TIC DISORDER
	30723	TOURETTE'S DISORDER
	30752	PICA
	30924	ADJUSTMENT DISORDER WITH ANXIETY
	30981	POSTTRAUMATIC STRESS DISORDER
	31210	UNDERSOCIALIZED CONDUCT DISORDER,UNAGGRESSIVE TYPE,UNSPECIFIED
	31239	OTHER DISORDERS OF IMPULSE CONTROL
	31389	OTHER EMOTIONAL DISTURBANCES OF CHILDHOOD OR ADOLESCENCE
	79921	NERVOUSNESS
	79922	IRRITABILITY
	79925	DEMORALIZATION AND APATHY
	79929	OTHER SIGNS AND SYMPTOMS INVOLVING EMOTIONAL STATE
	V6284	SUICIDAL IDEATION
**ADHD**	3142	HYPERKINETIC CONDUCT DISORDER OF CHILDHOOD
	3149	UNSPECIFIED HYPERKINETIC SYNDROME OF CHILDHOOD
	31400	ATTENTION DEFICIT DISORDER WITHOUT HYPERACTIVITY
	31401	ATTENTION DEFICIT DISORDER WITH HYPERACTIVITY
	V400	MENTAL AND BEHAVIORAL PROBLEMS WITH LEARNING
	V409	UNSPECIFIED MENTAL OR BEHAVIORAL PROBLEM
**DEVELOPMENTAL DISORDERS**	317	MILD INTELLECUTAL DISABILITIES
	317	MILD MENTAL RETARDATION
	319	UNSPECIFIED INTELLECTUAL DISABILITIES
	319	UNSPECIFIED MENTAL RETARDATION

	3152	OTHER SPECIFIC DEVELOPMENTAL LEARNING DIFFICULTIES
	3154	DEVELOPMENTAL COORDINATION DISORDER
	3158	OTHER SPECIFIED DELAYS IN DEVELOPMENT
	3159	UNSPECIFIED DELAY IN DEVELOPMENT
	7834	LACK OF EXPECTED NORMAL PHYSIOLOGICAL DEVELOPMENT
	7834	LACK OF EXPECTED NORMAL PHYSIOLOGICAL DEVELOPMENT IN CHILDHOOD
	31531	EXPRESSIVE LANGUAGE DISORDER
	31534	SPEECH AND LANGUAGE DEVELOPMENTAL DELAY DUE TO HEARING LOSS
	31539	OTHER DEVELOPMENTAL SPEECH DISORDER
	33183	MILD COGNITIVE IMPAIRMENT, SO STATED
	78340	LACK OF NORMAL PHYSIOLOGICAL DEVELOPMENT, UNSPECIFIED
**DEGENERATIVE DISORDERS**	330	CEREBRAL DEGENERATIONS USUALLY MANIFEST IN CHILDHOOD
	335	ANTERIOR HORN CELL DISEASE
	340	MULTIPLE SCLEROSIS
	3300	LEUKODYSTROPHY
	3308	OTHER SPECIFIED CEREBRAL DEGENERATIONS IN CHILDHOOD
	3313	COMMUNICATING HYDROCEPHALUS
	3314	OBSTRUCTIVE HYDROCEPHALUS
	3319	CEREBRAL DEGENERATION, UNSPECIFIED
	3348	OTHER SPINOCEREBELLAR DISEASES
	3350	WERDNIG-HOFFMANN DISEASE
	3360	SYRINGOMYELIA AND SYRINGOBULBIA
	3410	NEUROMYELITIS OPTICA
	3411	SCHILDER'S DISEASE
	3419	DEMYELINATING DISEASE OF CENTRAL NERVOUS SYSTEM, UNSPECIFIED
	3480	CEREBRAL CYSTS
	3590	CONGENITAL HEREDITARY MUSCULAR DYSTROPHY
	3591	HEREDITARY PROGRESSIVE MUSCULAR DYSTROPHY
	33189	OTHER CEREBRAL DEGENERATION
	33510	SPINAL MUSCULAR ATROPHY, UNSPECIFIED
	33522	PROGRESSIVE BULBAR PALSY
	33523	PSEUDOBULBAR PALSY
	34120	ACUTE (TRANSVERSE) MYELITIS NOS
	348891	CEREBRAL CALCIFICATION
	3313 2	POST HEMORRHAGIC HYDROCEPHALUS
**HEADACHE DISORDERS**	3469	MIGRAINE, UNSPECIFIED
	30781	TENSION HEADACHE
	34600	MIGRAINE WITH AURA WITHOUT MENTION OF INTRACTABLE MIGRAINE, WITHOU T MENTION OF STATUS MIGRAINOSUS
	34601	MIGRAINE WITH AURA, SO STATED,WITHOUT MENTION OF STATU.MIGRAINOSUS
	34620	VARIANTS OF MIGRAINE, WITHOUT INTRACTABLE MIGRAINE
	34630	HEMIPLEGIC MIGRAINE WITHOUT MENTION OF INTRACTABLE MIGRAINE, WITH OUT MENTION OF STATUS MIGRAINOSUS
	34670	CHRONIC MIGRAINE WITHOUT AURA WITHOUT MENTION OF INTRACTABLE MIGR AINE, WITHOUT MENTION OF STATUS MIGRAINOSUS
	34690	MIGRAINE, UNSPECIFIED, WITHOUT INTRACTABEL MIGRAINE

	34690	MIGRAINE, UNSPECIFIED, WITHOUT MENTION OF INTRACTABLE MIGRAINE WI THOUT MENTION OF STATUS MIGRAINOSUS
**MYOPATHIES**	352	DISORDERS OF OTHER CRANIAL NERVES
	3379	UNSPECIFIED DISORDER OF AUTONOMIC NERVOUS SYSTEM
	3510	BELL'S PALSY
	3518	OTHER FACIAL NERVE DISORDERS
	3519	FACIAL NERVE DISORDER, UNSPECIFIED
	3539	UNSPECIFIED NERVE ROOT AND PLEXUS DISORDER
	3542	LESION OF ULNAR NERVE
	3548	OTHER MONONEURITIS OF UPPER LIMB
	3549	MONONEURITIS OF UPPER LIMB, UNSPECIFIED
	3553	LESION OF LATERAL POPLITEAL NERVE
	3556	LESION OF PLANTAR NERVE
	3558	MONONEURITIS OF LOWER LIMB, UNSPECIFIED
	3559	MONONEURITIS OF UNSPECIFIED SITE
	3562	HEREDITARY SENSORY NEUROPATHY
	3564	IDIOPATHIC PROGRESSIVE POLYNEUROPATHY
	3568	OTHER SPECIFIED IDIOPATHIC PERIPHERAL NEUROPATHY
	3569	UNSPECIFIED IDIOPATHIC PERIPHERAL NEUROPATHY
	3570	ACUTE INFECTIVE POLYNEURITIS
	3571	POLYNEUROPATHY IN COLLAGEN VASCULAR DISEASE
	3572	POLYNEUROPATHY IN DIABETES
	3577	POLYNEUROPATHY DUE TO OTHER TOXIC AGENTS
	3588	OTHER SPECIFIED MYONEURAL DISORDERS
	3589	MYONEURAL DISORDERS, UNSPECIFIED
	3592	MYOTONIC DISORDERS
	3599	MYOPATHY, UNSPECIFIED
	33709	OTHER IDIOPATHIC PERIPHERAL AUTONOMIC NEUROPATHY
	33720	REFLEX SYMPATHETIC DYSTROPHY, UNSPECIFIED
	33721	REFLEX SYMPATHETIC DYSTROPHY OF UPPER LIMB
	33722	REFLEX SYMPATHETIC DYSTROPHY OF LOWER LIMB
	35781	CHRONIC INFLAMMATORY DEMYELINATING POLYNEURITIS
	35800	MYASTHENIA GRAVIS WITHOUT (ACUTE) EXACERBATION
**OTHER**	3383	NEOPLASM RELATED PAIN (ACUTE) (CHRONIC)
	3384	CHRONIC PAIN SYNDROME
	3482	BENIGN INTRACRANIAL HYPERTENSION
	3483	ENCEPHALOPATHY, UNSPECIFIED
	3483	ENCEPHALOPATHY, NOT ELSEWHERE CLASSIFIED
	3490	REACTION TO SPINAL OR LUMBAR PUNCTURE
	3492	DISORDERS OF MENINGES, NOT ELSEWHERE CLASSIFIED
	3499	UNSPECIFIED DISORDERS OF NERVOUS SYSTEM
	3561	PERONEAL MUSCULAR ATROPHY
	7802	SYNCOPE AND COLLAPSE
	7843	APHASIA
	30789	OTHER PSYCHALGIA

	33381	BLEPHAROSPASM
	33819	OTHER ACUTE PAIN
	33829	OTHER CHRONIC PAIN
	33903	EPISODIC PAROXYSMAL HEMICRANIA
	34830	ENCEPHALOPATHY, UNSPECIFIED
	34831	METABOLIC ENCEPHALOPATHY
	34881	TEMPORAL SCLEROSIS
	34889	OTHER CONDITIONS OF BRAIN
	34981	CEREBROSPINAL FLUID RHINORRHEA
	34989	OTHER SPECIFIED DISORDERS OF NERVOUS SYSTEM
	78093	MEMORY LOSS
	99701	CENTRAL NERVOUS SYSTEM COMPLICATION
	99709	OTHER NERVOUS SYSTEM COMPLICATIONS
	3488 1	CEREBRAL CALCIFICATION

Infectious morbidity

INFECTIOUS GROUP	DIAG. CODE	DIAGNOSIS DESCRIPTION
**URINARY TRACT INFECTIONS**	5901	ACUTE PYELONEPHRITIS
	5950	ACUTE CYSTITIS
	5959	CYSTITIS, UNSPECIFIED
	5970	URETHRAL ABSCESS
	5990	URINARY TRACT INFECTION, SITE NOT SPECIFIED
	59010	AC.PYELONEPHRITIS WITHOUT LESION OF RENAL MEDULLARY NECROSIS
	59080	PYELONEPHRITIS, UNSPECIFIED
	59581	CYSTITIS CYSTICA
	59589	OTHER SPECIFIED TYPES OF CYSTITIS
	59780	URETHRITIS, UNSPECIFIED
	59789	OTHER URETHRITIS
	V1302	PERSONAL HISTORY OF URINARY (TRACT) INFECTION
**GASTROINTESTINAL INFECTION**	008	INTESTINAL INFECTIONS DUE TO OTHER ORGANISMS
	0030	SALMONELLA GASTROENTERITIS
	0039	SALMONELLA INFECTION, UNSPECIFIED
	0040	SHIGELLA DYSENTERIAE
	0041	SHIGELLA FLEXNERI
	0042	SHIGELLA BOYDII
	0043	SHIGELLA SONNEI
	0048	OTHER SPECIFIED SHIGELLA INFECTIONS
	0049	SHIGELLOSIS, UNSPECIFIED
	0051	BOTULISM
	0059	FOOD POISONING, UNSPECIFIED
	0068	AMEBIC INFECTION OF OTHER SITES
	0069	AMEBIASIS, UNSPECIFIED
	0070	BALANTIDIASIS
	0071	GIARDIASIS
	0078	OTHER SPECIFIED PROTOZOAL INTESTINAL DISEASES
	0079	UNSPECIFIED PROTOZOAL INTESTINAL DISEASE
	0084	INTESTINAL INFECTION DUE TO OTHER SPECIFIED BACTERIA
	0085	BACTERIAL ENTERITIS, UNSPECIFIED
	0088	INTESTINAL INFECTION DUE TO OTHER ORGANISM,NOT ELSEW.CLASS.
	0090	INFECTIOUS COLITIS, ENTERITIS, & GASTROENTERITIS
	0090	INFECTIOUS COLITIS, ENTERITIS, AND GASTROENTERITIS
	0091	COLITIS,ENTERITIS,GASTROENTERITIS OF PRESUMED INF. ORIGIN
	0092	INFECTIOUS DIARRHEA
	129	INTESTINAL PARASITISM, UNSPECIFIED
	00842	INTESTINAL INFEC. DUE TO PSEUDOMONAS

	00843	INTESTINAL INFEC. DUE TO CAMPYLOBACTER
	00845	INTESTINAL INFEC. DUE TO CLOSTRIDIUM DIFFICILE
	00861	ENTERITIS DUE TO ROTAVIRUS
	00862	ENTERITIS DUE TO ADENOVIRUS
	00865	ENTERITIS DUE TO CALICIVIRUS
	00867	ENTERITIS DUE TO ENTEROVIRUS, N.E.C.
	00869	OTHER VIRAL ENTERITIS
	1274	ENTEROBIASIS
	1278	MIXED INTESTINAL HELMINTHIASIS
	1279	INTESTINAL HELMINTHIASIS, UNSPECIFIED
	1289	HELMINTH INFECTION, UNSPECIFIED
	5902	RENAL AND PERINEPHRIC ABSCESS
	V023	CARRIER OR SUSP.CARRIER OF OTHER GASTROINTESTINAL PATHOGENS
**CENTRAL NERVOUS SYSTEM (CNS) INFECTION**	0022	PARATYPHOID FEVER B
	0023	PARATYPHOID FEVER C
	048	OTHER ENTEROVIRUS DISEASES OF CENTRAL NERVOUS SYSTEM
	320	BACTERIAL MENINGITIS
	322	MENINGITIS OF UNSPECIFIED CAUSE
	325	PHLEBITIS AND THROMBOPHLEBITIS OF INTRACRANIAL VENOUS SINUSES
	326	LATE EFFECTS OF INTRACRANIAL ABSCESS OR PYOGENIC INFECTION
	0470	MENINGITIS DUE TO COXSACKIE VIRUS
	0471	MENINGITIS DUE TO ECHO VIRUS
	0478	OTHER SPECIFIED VIRAL MENINGITIS
	0479	UNSPECIFIED VIRAL MENINGITIS
	0491	NON-ARTHOPOD-BORNE MENINGITIS DUE TO ADENOVIRUS
	0499	UNSP.NON-ARTHROPOD-BORNE VIRAL DIS.OF CENTRAL NERVOUS SYSTEM
	0630	RUSSIAN SPRING-SUMMER (TAIGA) ENCEPHALITIS
	3200	HEMOPHILUS MENINGITIS
	3201	PNEUMOCOCCAL MENINGITIS
	3202	STREPTOCOCCAL MENINGITIS
	3203	STAPHYLOCOCCAL MENINGITIS
	3207	MENINGITIS IN OTHER BACTERIAL DISEASES CLASSIFIED ELSEWHERE
	3208	MENINGITIS DUE TO OTHER SPECIFIED BACTERIA
	3209	MENINGITIS DUE TO UNSPECIFIED BACTERIUM
	3229	MENINGITIS, UNSPECIFIED
	3236	POSTINFECTIOUS ENCEPHALITIS
	3238	OTHER CAUSES OF ENCEPHALITIS
	3238	OTHER CAUSES OF ENCEPHALITIS, MYELITIS AND ENCEPHALOMYELITIS
	3239	UNSPECIFIED CAUSE OF ENCEPHALITIS
	3239	UNSPECIFIED CAUSE OF ENCEPHALITIS, MYELITIS,AND ENCEPHALOMYELITIS
	3240	INTRACRANIAL ABSCESS
	3241	INTRASPINAL ABSCESS

	3249	INTRACRANIAL AND INTRASPINAL ABSCESS OF UNSPECIFIED SITE
	06641	WEST NILE FEVER WITH ENCEPHALITIS
	32082	MENINGITIS DUE TO GRAM-NEGATIVE
	32089	MENINGITIS DUE TO OTHER SPECIFIED BACTERIA
	32361	INFECTIOUS ACUTE DISSEMINATED ENCEPHALOMYELITIS (ADEM)
	32381	OTHER CAUSES OF ENCEPHALITIS AND ENCEPHLOMYELITIS
	32382	OTHER CAUSES OF MYELITIS
	0360	MENINGOCOCCAL MENINGITIS
**EXTRAINTESTINAL SALMONELLA INFECTION**	0031	SALMONELLA SEPTICEMIA
	0038	OTHER SPECIFIED SALMONELLA INFECTIONS
	00321	SALMONELLA MENINGITIS
	00323	SALMONELLA ARTHRITIS
	00329	OTHER LOCALIZED SALMONELLA INFECTIONS
**H. PYLORI INFECTION**	04186	HELICOBACTER PYLORI (H. PYLORI) INFECTION
**TUBERCULOSIS INFECTION**	0119	UNSPECIFIED PULMONARY TUBERCULOSIS
	01090	PRIM. TB. INFEC., UNSP.TYPE, UNSP.EXAMINATION
	01120	TB. OF LUNG + CAVITATION, UNSP. EXAMINATION
	01190	UNSP. PULMONARY TB., UNSP. EXAMINATION
	01194	UNSP. PULMONARY TB., FOUND BY BACTERIAL CULTURE
	01304	TB. MENINGITIS, FOUND BY BACTERIAL CULTURE
	01311	TUBERCULOMA OF MENINGES, BACT/HISTOL. EXAM. NOT DONE
	01394	UNSP. TB. OF C.N.S. FOUND BY BACTERIAL CULTURE
	01404	TB. PERITONITIS, FOUND BY BACTERIAL CULTURE
	01485	OTHER TB. INTESTINES, CONFIRMED HISTOLOGICALLY
	V1201	PERSONAL HISTORY OF TUBERCULOSIS
**OPPORUNISTIC RARE INFECTIONS**	022	ANTHRAX
	0073	INTESTINAL TRICHOMONIASIS
	0074	CRYPTOSPORIDIOSIS
	0075	CYCLOSPORIASIS
	0082	INTESTINAL INFEC. DUE TO AEROBACTER AEROGENES
	0205	PNEUMONIC PLAGUE, UNSPECIFIED
	0209	PLAGUE, UNSPECIFIED
	0783	CAT-SCRATCH DISEASE
	0810	MURINE (ENDEMIC) TYPHUS
	0820	SPOTTED FEVERS
	0830	Q FEVER
	0838	OTHER SPECIFIED RICKETTSIOSES
	0839	RICKETTSIOSIS, UNSPECIFIED
	00841	INTESTINAL INFEC. DUE TO STAPHYLOCOCCUS
	0846	MALARIA, UNSPECIFIED
	0859	LEISHMANIASIS, UNSPECIFIED
	0879	RELAPSING FEVER, UNSPECIFIED

	0909	CONGENITAL SYPHILIS, UNSPECIFIED
	0940	TABES DORSALIS
	0980	GONOCOCCAL INFEC.,ACUTE, OF LOWER GENITOURINARY TRACT
	1000	LEPTOSPIROSIS ICTEROHEMORRHAGICA
	1160	BLASTOMYCOSIS
	1173	ASPERGILLOSIS
	1175	CRYPTOCOCCOSIS
	1177	ZYGOMYCOSIS (PHYCOMYCOSIS OR MUCORMYCOSIS)
	1179	OTHER AND UNSPECIFIED MYCOSES
	1209	SCHISTOSOMIASIS, UNSPECIFIED
	1225	ECHINOCOCCUS MULTILOCULARIS INFECTION OF LIVER
	1228	ECHINOCOCCOSIS, UNSPECIFIED, OF LIVER
	1229	ECHINOCOCCOSIS, OTHER AND UNSPECIFIED
	1270	ASCARIASIS
	1363	PNEUMOCYSTOSIS
	1369	UNSP. INFECTIOUS & PARASITIC DISEASES
	1369	UNSPECIFIED INFECTIOUS AND PARASITIC DISEASES
	04183	OTHER CLOSTRIDIUM PERFRINGENS INFECTION
	08881	LYME DISEASE (ERYTHEMA CHRONICUM MIGRANS)
	08882	BABESIOSIS
	09840	GONOCOCCAL CONJUNCTIVITIS (NEONATORUM)
	09882	GONOCOCCAL MENINGITIS
	09886	GONOCOCCAL PERITONITIS
**BRUCELLOSIS**	0239	BRUCELLOSIS, UNSPECIFIED
	0269	UNSPECIFIED RAT-BITE FEVER
	0270	LISTERIOSIS
	0312	DISSEMINATED DISEASE DUE TO OTHER MYCOBACTERIA
	0319	UNSPECIFIED DISEASES DUE TO MYCOBACTERIA
	03283	DIPHTHERITIC PERITONITIS
**INFECTIONS NOS**	0418	OTHER SPEC.BACTERIAL INF;IN CONDIT.CLASS.ELSEWHERE,UNSP.SITE
	0419	UNSP. BACTERIAL INFECTION, UNSP. SITE
	0419	UNSPEC.BACTERIAL INF;IN CONDIT. CLASSIF.ELSEWHERE,UNSP.SITE
	04189	OTHER SPECIFIED BACTERIA INFECTION
	99762	INFECTION (CHRONIC) OF AMPUTATION STUMP
	V091	INFECTION WITH MICROORGAN. RESISTANT TO CEPHALOSPORINS/B-LACTAM ANTIBIOTICS
	V0980	INFECTION WITH MICROORGAN. RESISTANT TO OTHER SPEC. DRUGS, WITHOUT MENTION OF RESISTANCE TO MULTIPLE DRUGS
	V0991	INFECTION WITH DRUG-RESISTANT MICROORGAN.,UNSPEC. DRUG RESISTANCE NOS, WITH MULTIPLE DRUG RESISTANCE
**PERTUSSIS**	0330	WHOOPING COUGH DUE TO BORDETELLA PERTUSSIS (B. PERTUSSIS)
	0331	WHOOPING COUGH DUE TO BORDETELLA PARAPERTUSSIS
	0339	WHOOPING COUGH, UNSPECIFIED ORGANISM
	034	STREPTOCOCCAL SORE THROAT AND SCARLET FEVER

**STREPTOCOCCUS AND STAPHYLOCOCCUS INFECTIONS**	035	ERYSIPELAS
	0340	STREPTOCOCCAL SORE THROAT
	0341	SCARLET FEVER
	0410	STREPTOCOCCUS INFECT.IN CONDITION CLASSIF.ELSEWHERE;UNSP.SITE
	0411	STAPHYLOCOCCUS INFECT.IN CONDIT.CLASSIF.ELSEWHERE,UNSP.SITE
	0411	STAPHYLOCOCCUS INFECTION, UNSP. SITE
	0412	PNEUMOCOCCUS INFECT.IN CONDITION CLASSIF.ELSEWHERE;UNSP.SITE
	0412	PNEUMOCOCCUS INFECTION, UNSP. SITE
	04100	STREPTOCOCCUS INFEC., UNSP.
	04101	STREPTOCOCCUS INFEC., GROUP A
	04101	STREPTOCOCCUS INFEC., GROUP A (ADDITIONAL CODE)
	04102	STREPTOCOCCUS INFEC., GROUP B
	04103	STREPTOCOCCUS INFEC., GROUP C
	04104	STREPTOCOCCUS INFEC., GROUP D (ENTEROCOCCUS)
	04105	STREPTOCOCCUS INFEC., GROUP G
	04109	OTHER STREPTOCOCCUS INFEC.
	04110	STAPHYLOCOCCUS INFECTION, UNSP.
	04111	METHICILLIN SUSCEPTIBLE STAPHYLOCOCCUS AUREUS
	04111	STAPHYLOCOCCUS AUREUS INFEC.
	04111	STAPHYLOCOCCUS AUREUS INFEC. (ADDITIONAL CODE)
	04112	METHICILLIN RESISTANT STAPHYLOCOCCUS AUREUS
	04119	OTHER STAPHYLOCOCCUS INFECTION
	V0252	CARRIER OR SUSP. CARRIER OF OTHER STREPTOCOCCUS
	V0259	CARRIER/SUSP. CARRIER OF OTHER SPECIFIED BACTERIAL DIS.
**MENINGOCOCCAL INFECTION**	0362	MENINGOCOCCEMIA
	0369	MENINGOCOCCAL INFECTION, UNSPECIFIED
	03682	MENINGOCOCCAL ARTHROPATHY
	03689	OTHER SPECIFIED MENINGOCOCCAL INFECTIONS
**SEPSIS**	0380	STREPTOCOCCAL SEPTICEMIA
	0381	STAPHYLOCOCCAL SEPTICEMIA
	0382	PNEUMOCOCCAL SEPTICEMIA
	0383	SEPTICEMIA DUE TO ANAEROBES
	0388	OTHER SPECIFIED SEPTICEMIAS
	0389	UNSPECIFIED SEPTICEMIA
	03810	STAPHYLOCOCCAL SPETICEMIA, UNSP.
	03811	METHICILLIN SUSCEPTIBLE STAPHYLOCOCCUS AUREUS SEPTICEMIA
	03811	STAPHYLOCOCCUS AUREUS SEPTICEMIA
	03812	METHICILLIN RESISTANT STAPHYLOCOCCUS AUREUS SEPTICEMIA
	03819	OTHER STAPHYLOCOCCAL SEPTICEMIA
	03840	SEPTICEMIA DUE TO GRAM-NEGATIVE ORGANISM, UNSPECIFIED
	03841	SEPTICEMIA DUE TO HEMOPHILUS INFLUENZAE (H. INFLUENZAE)
	03842	SEPTICEMIA DUE TO ESCHERICHIA COLI (E. COLI)

	03843	SEPTICEMIA DUE TO PSEUDOMONAS
	03849	OTHER SEPTICEMIA DUE TO GRAM-NEGATIVE ORGANISMS
	038491	KLEBSIELLA SEPTICEMIA
	0388 1	CANDIDA SEPTICEMIA
	0388 2	ENTEROCOCCUS SEPTICEMIA
	0388 3	ACITINOBACTER SEPTICEMIA
**GRAM-NEGATIVE INFECTION**	0413	FRIEDLANDER'S BACILLUS, UNSP. SITE
	0413	FRIEDLANDER'S BACILLUS;CONDITION CLASSIF.ELSEWHERE,UNSP.SITE
	0413	KLEBSIELLA PNEUMONIAE
	0414	ESCHERICHIA COLI (E. COLI), UNSP. SITE
	0414	ESCHERICHIA COLI (E. COLI), UNSP. SITE (ADDITIONAL CODE)
	0414	ESCHERICHIA COLI(E. COLI),CONDITI.CLASSIF.ELSEWHERE,UNSP.SITE
	0415	HEMOPHILUS INFLUENZAE IN CONDIT.CLASSIF.ELSEWHERE,UNSP.SITE
	0415	HEMOPHILUS INFLUENZAE, UNSP. SITE (H.INFLUENZAE)
	0416	PROTEUS (MIRABILIS,MORGANII), UNSP. SITE
	0416	PROTEUS(MIRABILIS,MORGANII)CONDIT,CLASSIF.ELSEWHERE,UNSP.SITE
	0417	PSEUDOMONAS INFEC., UNSP. SITE
	0417	PSEUDOMONAS INFECT.IN CONDIT.CLASSIF.ELSEWHERE,UNSPEC.SITE
	04185	OTHER GRAM-NEGATIVE ORGANISMS INFECTION
**HUMAN IMMUNODEFICIENCY VIRUS (HIV)**	042	HUMAN IMMUNODEFIC. VIRUS (HIV) DIS. /AIDS
	V08	ASYMPTOMATIC H.I.V INFECTION STATUS
**HERPES, VARICELLA**	0521	VARICELLA (HEMORRHAGIC) PNEUMONITIS
	0527	CHICKENPOX WITH OTHER SPECIFIED COMPLICATIONS
	0528	CHICKENPOX WITH UNSPECIFIED COMPLICATION
	0529	VARICELLA WITHOUT MENTION OF COMPLICATION
	0539	HERPES ZOSTER WITHOUT MENTION OF COMPLICATION
	0540	ECZEMA HERPETICUM
	0542	HERPETIC GINGIVOSTOMATITIS
	0543	HERPETIC MENINGOENCEPHALITIS
	0546	HERPETIC WHITLOW
	0549	HERPES SIMPLEX WITHOUT MENTION OF COMPLICATION
	0559	MEASLES WITHOUT MENTION OF COMPLICATION
	0569	RUBELLA WITHOUT MENTION OF COMPLICATION
	0570	ERYTHEMA INFECTIOSUM (FIFTH DISEASE)
	0578	OTHER SPECIFIED VIRAL EXANTHEMATA
	0579	VIRAL EXANTHEM, UNSPECIFIED
	0723	MUMPS PANCREATITIS
	0729	MUMPS WITHOUT MENTION OF COMPLICATION
	0740	HERPANGINA
	0743	HAND, FOOT, AND MOUTH DISEASE
	05311	GENICULATE HERPES ZOSTER
	05319	HERPES ZOSTER + OTHER NERVOUS SYSTEM COMPLICATIONS

	05320	HERPES ZOSTER DERMATITIS OF EYELID
	05329	HERPES ZOSTER + OTHER OPHTHALMIC COMPLICATIONS
	05410	GENITAL HERPES, UNSPECIFIED
	05440	HERPES SIMPLEX + UNSP. OPHTHALMIC COMPLICATION
	05441	HERPES SIMPLEX DERMATITIS OF EYELID
	05443	HERPES SIMPLEX DISCIFORM KERATITIS
	05449	HERPES SIMPLEX WITH OTHER OPHTHALMIC COMPLICATIONS
	05472	HERPES SIMPLEX MENINGITIS
	05479	HERPES SIMPLEX + OTHER SPEC. COMPLICATIONS
	05810	ROSEOLA INFANTUM, UNSPECIFIED
	05829	OTHER HUMAN HERPESVIRUS ENCEPHALITIS
	0784	FOOT AND MOUTH DISEASE
**HEPATITIS**	0701	VIRAL HEPATITIS A WITHOUT HEPATIC COMA
	0701	VIRAL HEPATITIS A WITHOUT MENTION OF HEPATIC COMA
	0709	UNSP. VIRAL HEPATITIS WITHOUT HEPATIC COMA
	0709	UNSPECIFIED VIRAL HEPATITIS WITHOUT MENTION OF HEPATIC COMA
	07030	VIRAL HEPATITIS B WITHOUT HEPATIC COMA & HEPATITIS DELTA -92
	07030	VIRAL HEPATITIS B WITHOUT HEPATIC COMA,AC/UNSP.WITHOUT HEP. DELTA
	07032	VIRAL HEPATITS B WITHOUT HEPATIC COMA,CHR. WITHOUT HEPATITIS DELT
	V0261	HEPATITIS B CARRIER
	V0262	HEPATITIS C CARRIER
**EBV, CMV**	075	INFECTIOUS MONONUCLEOSIS
	0785	CYTOMEGALIC INCLUSION DISEASE
	0785	CYTOMEGALOVIRAL DISEASE
**WARTS**	0780	MOLLUSCUM CONTAGIOSUM
	0781	VIRAL WARTS
	07810	VIRAL WARTS, UNSPECIFIED
	07811	CONDYLOMA ACUMINATUM
	07812	PLANTAR WART
	07819	OTHER SPECIFIED VIRAL WARTS
**VIRAL INFECTION NOS**	0790	ADENOVIRUS INF.IN CONDITIONS CLASSIF.ELSEWHERE,UNSP.SITE
	0790	ADENOVIRUS INFECTION, UNSP. SITE
	0791	ECHO VIRUS INFECTION, UNSP. SITE
	0792	COXSACKIE VIRUS INFECTION, UNSP. SITE
	0793	RHINOVIRUS INFECTION, UNSP. SITE
	0798	OTHER SPECIFIED VIRAL INFECTION CLASSIF.ELSEWHERE,UNSP.SITE
	0799	UNSP. VIRAL & CHLANYDIAL INFECTION
	0799	UNSP.VIRAL INFECT.IN CONDITIONS CLASSIF.ELSEWHERE,UNSP.SITE
	07889	OTHER SPECIFIED DISEASES DUE TO VIRUSES
	07889	OTHER SPECIFIED DISEASES DUE TO VIRUSES AND CHLAMYDIAE
	07950	RETROVIRUS, UNSP.,UNSP. SITE
	07959	OTHER SPECIFIED RETROVIRUS

	07989	OTHER SPEC. VIRAL INFECTION
	07999	UNSP. VIRAL INFECTION
	07999	UNSP. VIRAL INFECTION (ADDITIONAL CODE)
**RSV INFECTION**	0796	RESPIRATORY SYNCYTIAL VIRUS (RSV)
**FUNGAL INFECTION**	1100	DERMATOPHYTOSIS OF SCALP AND BEARD
	1101	DERMATOPHYTOSIS OF NAIL
	1103	DERMATOPHYTOSIS OF GROIN AND PERIANAL AREA
	1104	DERMATOPHYTOSIS OF FOOT
	1105	DERMATOPHYTOSIS OF THE BODY
	1109	DERMATOPHYTOSIS OF UNSPECIFIED SITE
	1110	PITYRIASIS VERSICOLOR
	1118	OTHER SPECIFIED DERMATOMYCOSES
	1119	DERMATOMYCOSIS, UNSPECIFIED
	1120	CANDIDIASIS OF MOUTH
	1121	CANDIDIASIS OF VULVA AND VAGINA
	1122	CANDIDIASIS OF OTHER UROGENITAL SITES
	1123	CANDIDIASIS OF SKIN AND NAILS
	1124	CANDIDIASIS OF LUNG
	1129	CANDIDIASIS OF UNSPECIFIED SITE
	11281	CANDIDAL ENDOCARDITIS
	11282	CANDIDAL OTITIS EXTERNA
	11284	CANDIDAL ESOPHAGITIS
	11289	OTHER CANDIDIASIS OF OTHER SPECIFIED SITES
**OTITIS**	382	SUPPURATIVE AND UNSPECIFIED OTITIS MEDIA
	3813	OTHER AND UNSPECIFIED CHRONIC NONSUPPURATIVE OTITIS MEDIA
	3814	NONSUPPURATIVE OTITIS MEDIA, NOT SPECIFIED AS ACUTE OR CHRONIC
	3819	UNSPECIFIED EUSTACHIAN TUBE DISORDER
	3820	ACUTE SUPPURATIVE OTITIS MEDIA
	3821	CHRONIC TUBOTYMPANIC SUPPURATIVE OTITIS MEDIA
	3823	UNSPECIFIED CHRONIC SUPPURATIVE OTITIS MEDIA
	3824	UNSPECIFIED SUPPURATIVE OTITIS MEDIA
	3829	UNSPECIFIED OTITIS MEDIA
	3831	CHRONIC MASTOIDITIS
	3839	UNSPECIFIED MASTOIDITIS
	3841	CHRONIC MYRINGITIS WITHOUT MENTION OF OTITIS MEDIA
	3849	UNSPECIFIED DISORDER OF TYMPANIC MEMBRANE
	38001	ACUTE PERICHONDRITIS OF PINNA
	38002	CHRONIC PERICHONDRITIS OF PINNA
	38003	CHONDRITIS OF PINNA
	38010	INFECTIVE OTITIS EXTERNA, UNSPECIFIED
	38015	CHRONIC MYCOTIC OTITIS EXTERNA
	38022	OTHER ACUTE OTITIS EXTERNA

	38023	OTHER CHRONIC OTITIS EXTERNA
	38100	ACUTE NONSUPPURATIVE OTITIS MEDIA, UNSPECIFIED
	38101	ACUTE SEROUS OTITIS MEDIA
	38104	ACUTE ALLERGIC SEROUS OTITIS MEDIA
	38110	CHRONIC SEROUS OTITIS MEDIA, SIMPLE OR UNSPECIFIED
	38119	OTHER CHRONIC SEROUS OTITIS MEDIA
	38129	OTHER CHRONIC MUCOID OTITIS MEDIA
	38200	AC.SUPPURAT.OTITIS MEDIA WITHOUT SPONTAN.RUPTURE OF EARDRUM
	38201	ACUTE SUPPURAT.OTITIS MEDIA WITH SPONTAN.RUPTURE OF EARDRUM
	38300	ACUTE MASTOIDITIS WITHOUT COMPLICATIONS
	38301	SUBPERIOSTEAL ABSCESS OF MASTOID
	38330	POSTMASTOIDECTOMY COMPLICATION, UNSPECIFIED
	38389	OTHER DISORDERS OF MASTOID
	38400	ACUTE MYRINGITIS, UNSPECIFIED
	38401	BULLOUS MYRINGITIS
	38420	PERFORATION OF TYMPANIC MEMBRANE, UNSPECIFIED
	38482	ATROPHIC NONFLACCID TYMPANIC MEMBRANE
	38500	TYMPANOSCLEROSIS, UNSPECIFIED AS TO INVOLVEMENT
	38510	ADHESIVE MIDDLE EAR DISEASE, UNSPECIFIED AS TO INVOLVEMENT
	38530	CHOLESTEATOMA, UNSPECIFIED
	38531	CHOLESTEATOMA OF ATTIC
	38532	CHOLESTEATOMA OF MIDDLE EAR
	38630	LABYRINTHITIS, UNSPECIFIED
	38635	VIRAL LABYRINTHITIS
**UPPER RESPIRATORY INFECTION**	460	ACUTE NASOPHARYNGITIS (COMMON COLD)
	462	ACUTE PHARYNGITIS
	463	ACUTE TONSILLITIS
	464	ACUTE LARYNGITIS AND TRACHEITIS
	465	ACUTE UPPER RESPIRATORY INFECTIONS OF MULTIPLE OR UNSP.SITES
	475	PERITONSILLAR ABSCESS
	4610	ACUTE MAXILLARY SINUSITIS
	4611	ACUTE FRONTAL SINUSITIS
	4612	ACUTE ETHMOIDAL SINUSITIS
	4613	ACUTE SPHENOIDAL SINUSITIS
	4618	OTHER ACUTE SINUSITIS
	4619	ACUTE SINUSITIS, UNSPECIFIED
	4640	ACUTE LARYNGITIS
	4644	CROUP
	4650	ACUTE LARYNGOPHARYNGITIS
	4658	ACUTE UPPER RESPIRATORY INFECTIONS OF OTHER MULTIPLE SITES
	4659	ACUTE UPPER RESPIRATORY INFECTIONS OF UNSPECIFIED SITE
	4720	CHRONIC RHINITIS

	4730	CHRONIC MAXILLARY SINUSITIS
	4731	CHRONIC FRONTAL SINUSITIS
	4732	CHRONIC ETHMOIDAL SINUSITIS
	4733	CHRONIC SPHENOIDAL SINUSITIS
	4738	OTHER CHRONIC SINUSITIS
	4739	UNSPECIFIED SINUSITIS (CHRONIC)
	4741	HYPERTROPHY OF TONSILS AND ADENOIDS
	4742	ADENOID VEGETATIONS
	4748	OTHER CHRONIC DISEASE OF TONSILS AND ADENOIDS
	4749	UNSPECIFIED CHRONIC DISEASE OF TONSILS AND ADENOIDS
	4870	INFLUENZA WITH PNEUMONIA
	4871	INFLUENZA WITH OTHER RESPIRATORY MANIFESTATIONS
	4878	INFLUENZA WITH OTHER MANIFESTATIONS
	4880	INFLUENZA DUE TO IDENTIFIED AVIAN INFLUENZA VIRUS
	4881	INFLUENZA DUE TO IDENTIFIED NOVEL H1N1 INFLUENZA VIRUS
	46400	ACUTE LARYNGITIS WITHOUT MENTION OF OBSTRUCTION
	46410	ACUTE TRACHEITIS WITHOUT MENTION OF OBSTRUCTION
	46420	ACUTE LARYNGOTRACHEITIS WITHOUT MENTION OF OBSTRUCTION
	46430	ACUTE EPIGLOTTITIS WITHOUT MENTION OF OBSTRUCTION
	46450	SUPRAGLOTTITIS WITHOUT MENTION OF OBSTRUCTION
	47400	CHRONIC TONSILLITIS
	47401	CHRONIC ADENOIDITIS
	47410	HYPERTROPHY OF TONSIL WITH ADENOIDS
	47411	HYPERTROPHY OF TONSILS ALONE
	47412	HYPERTROPHY OF ADENOIDS ALONE
	47822	PARAPHARYNGEAL ABSCESS
	47824	RETROPHARYNGEAL ABSCESS
**BRONCHIOLITIS**	466	ACUTE BRONCHITIS AND BRONCHIOLITIS
	480	VIRAL PNEUMONIA
	490	BRONCHITIS, NOT SPECIFIED AS ACUTE OR CHRONIC
	4660	ACUTE BRONCHITIS
	4661	ACUTE BRONCHIOLITIS
	4800	PNEUMONIA DUE TO ADENOVIRUS
	4801	PNEUMONIA DUE TO RESPIRATORY SYNCYTIAL VIRUS
	4802	PNEUMONIA DUE TO PARAINFLUENZA VIRUS
	4808	PNEUMONIA DUE TO OTHER VIRUS NOT ELSEWHERE CLASSIFIED
	4809	VIRAL PNEUMONIA, UNSPECIFIED
	4841	PNEUMONIA IN CYTOMEGALIC INCLUSION DISEASE
	4918	OTHER CHRONIC BRONCHITIS
	46611	AC. BRONCHIOLITIS DUE TO RESPIRATORY SYNCYTIAL VIRUS (RSV)
	46619	AC. BRONCHIOLITIS DUE TO OTHER INFECTIOUS ORGANISMS
	49121	OBSTRUCTIVE CHR. BRONCHITIS WITH(ACUTE)EXACERBATION

	V1261	PERSONAL HISTORY OF PNEUMONIA (RECURRENT)
**PNEUMONIA**	481	PNEUMOCOCCAL PNEUMONIA
	481	PNEUMOCOCCAL PNEUMONIA (STREPTOCOCUUS PNEUMONIAE PNEUMONIA)
	483	PNEUMONIA DUE TO OTHER SPECIFIED ORGANISM
	4820	PNEUMONIA DUE TO KLEBSIELLA PNEUMONIAE
	4821	PNEUMONIA DUE TO PSEUDOMONAS
	4822	PNEUMONIA DUE TO HEMOPHILUS INFLUENZAE (H. INFLUENZAE)
	4823	PNEUMONIA DUE TO STREPTOCOCCUS
	4829	BACTERIAL PNEUMONIA, UNSPECIFIED
	4830	PNEUMONIA DUE TO MYCOPLASMA PNEUMONIAE
	4831	PNEUMONIA DEU TO CHLAMYDIA
	4838	PNEUMONIA DUE TO OTHER SPECIFIED ORGANISM
	48230	PNEUMONIA DUE TO STREPTOCOCCUS, UNSPECIFIED
	48231	PNEUMONIA DUE TO STREPTOCOCCUS, GROUP A
	48241	PNEUMONIA DUE TO STAPHYLOCOCCUS AUREUS
	99590	SYSTEMIC INFLAMMATORY RESPONSE SYNDROME, UNSPECIFIED
	99591	SEPSIS
	99591	SYSTEMIC INFLAMMATORY RESPONSE SYNDROME DUE TO INFECTIOUS PROCESS WITHOUT ORGAN DYSFUNCTION
	99592	SEVERE SEPSIS
	99592	SYSTEMIC INFLAMMATORY RESPONSE SYNDROME DUE TO INFECTIOUS PROCESS WITH ORGAN DYSFUNCTION
**OTHER**	101	VINCENT'S ANGINA
	0400	GAS GANGRENE
	0990	CHANCROID
	0993	REITER'S DISEASE
	0999	VENEREAL DISEASE, UNSPECIFIED
	1236	HYMENOLEPIASIS
	1307	TOXOPLASMOSIS OF OTHER SPECIFIED SITES
	1309	TOXOPLASMOSIS, UNSPECIFIED
	1320	PEDICULUS CAPITIS (HEAD LOUSE)
	1322	PHTHIRUS PUBIS (PUBIC LOUSE)
	1323	MIXED PEDICULOSIS INFESTATION
	1329	PEDICULOSIS, UNSPECIFIED
	1330	SCABIES
	1340	MYIASIS
	04082	TOXIC SHOCK SYNDROME
	04089	OTHER SPECIFIED BACTERIAL DISEASES
	04184	OTHER ANAEROBES INFECTION
	05442	DENDRITIC KERATITIS
	07988	OTHER SPEC. CHLAMYDIAL INFECTION
	07998	UNSP. CHLAMYDIAL INFECTION

Respiratory morbidity

GROUPS	DIAG. CODE	DIAGNOSIS DESCRIPTION
**ASTHMA**	49300	EXTRINSIC ASTHMA, UNSPECIFIED
	49320	CHR. OBSTRUCTIVE ASTHMA, UNSPECIFIED
	49321	CHR. OSBTRUCTIVE ASTHMA WITH STATUS ASTHMATICUS
	49390	ASTHMA, UNSPECIFIED
	49390	ASTHMA, UNSPECIFIED TYPE, WITHOUT MENTION OF STATUS ASTHMATICUS
	49390	ASTHMA, UNSPECIFIED TYPE, WITHOUT MENTION OF STATUS ASTHMATICUS OR ACUTE EXACERBATION OR UNSPECIFIED
	49391	ASTHMA, UNSPECIFIED TYPE, WITH STATUS ASTHMATICUS
	49392	UNSPECIFIED ASTHMA WITH (ACUTE) EXACERBATION
	496	CHRONIC AIRWAY OBSTRUCTION, NOT ELSEWHERE CLASSIFIED
**STRUCTURAL - EMPHYSEMA**	4920	EMPHYSEMATOUS BLEB
	4928	OTHER EMPHYSEMA
	5100	EMPYEMA WITH FISTULA
	5109	EMPYEMA WITHOUT MENTION OF FISTULA
	5181	INTERSTITIAL EMPHYSEMA
**BRONCHIECTASIS - FIBROSIS - HEMOSIDEROSIS**	494	BRONCHIECTASIS
	4940	BRONCHIECTESIS WITHOUT ACUTE EXACERBATION
	4941	BRONCHIECTASIS WITH ACUTE EXACERBATION
	4959	UNSPECIFIED ALLERGIC ALVEOLITIS AND PNEUMONITIS
	515	POSTINFLAMMATORY PULMONARY FIBROSIS
	5160	PULMONARY ALVEOLAR PROTEINOSIS
	5161	IDIOPATHIC PULMONARY HEMOSIDEROSIS
**PNEUMONITIS**	5070	PNEUMONITIS DUE TO INHALATION (FOOD,VOMITUS,OR N.O.S.)
	5070	PNEUMONITIS DUE TO INHALATION OF FOOD OR VOMITUS
	5071	PNEUMONITIS DUE TO INHALATION OF OILS AND ESSENCES
	5078	PNEUMONITIS DUE TO OTHER SOLIDS AND LIQUIDS
	5130	ABSCESS OF LUNG
	5168	OTHER SPECIFIED ALVEOLAR AND PARIETOALVEOLAR PNEUMONOPATHIES
	5183	PULMONARY EOSINOPHILIA
	5192	MEDIASTINITIS
	5193	OTHER DISEASES OF MEDIASTINUM, NOT ELSEWHERE CLASSIFIED
**PLEURAL**	5110	PLEURISY WITHOUT MENTION OF EFFUSION OR CURRENT TUBERCULOSIS
	5118	OTHER SPECIFIED FORMS OF PLEURAL EFFUSION, EXCEPT TUBERCULOUS
	5119	UNSPECIFIED PLEURAL EFFUSION
	5120	SPONTANEOUS TENSION PNEUMOTHORAX
	5128	OTHER SPONTANEOUS PNEUMOTHORAX
	51181	MALIGNANT PLEURAL EFFUSION
	51189	OTHER SPECIFIED FORMS OF EFFUSION, EXCEPT TUBERCULOUS
**OBSTRUCTIVE SLEEP APNEA (OSA)**	32723	OBSTRUCTIVE SLEEP APNEA (ADULT)(PEDIATRIC)
	32727	CENTRAL SLEEP APNEA IN CONDITIONS CLASSIFIED ELSEWHERE
	78051	INSOMNIA WITH SLEEP APNEA
	78051	INSOMNIA WITH SLEEP APNEA, UNSPECIFIED
	78057	OTHER AND UNSPECIFIED SLEEP APNEA

	78057	UNSPECIFIED SLEEP APNEA
**OTHER**	514	PULMONARY CONGESTION AND HYPOSTASIS
	5178	LUNG INVOLVEMENT IN OTHER DISEASES CLASSIFIED ELSEWHERE
	518	OTHER DISEASES OF LUNG
	5180	PULMONARY COLLAPSE
	5185	PULMONARY INSUFFICIENCY FOLLOWING TRAUMA AND SURGERY
	5186	ALLERGIC BRONCHOPULMONARY ASPERGILLOSIS
	51883	CHRONIC RESPIRATORY FAILURE
	51900	TRACHEOSTOMY COMPLICATION, UNSP.
	51909	OTHER TRACHEOSTOMY COMPLICATIONS
	5191	OTHER DISEASES OF TRACHEA AND BRONCHUS, NOT ELSEWHERE CLASSIF.
	5198	OTHER DISEASES OF RESPIRATORY SYSTEM, NOT ELSEWHERE CLASSIFIED
	5199	UNSPECIFIED DISEASE OF RESPIRATORY SYSTEM
	51911	ACUTE BRONCHOSPASM
	51919	OTHER DISEASES OF TRACHEA BRONCHUS
	786	SYMPTOMS INVOLVING RESPIRATORY SYSTEM & OTHER CHEST SYMPTOMS
	78607	WHEEZING
	78609	OTHER DYSPNEA AND RESPIRATORY ABNORMALITY
	7990	ASPHYXIA
	7990	ASPHYXIA AND HYPOXEMIA
	9973	RESPIRATORY COMPLICATIONS, NOT ELSEWHERE CLASSIFIED
	99739	OTHER RESPIRATORY COMPLICATIONS

Endocrine morbidity

GROUPS	DIAG. CODE	DIAGNOSIS DESCRIPTION
THYROID DISORDERS	243	CONGENITAL HYPOTHYROIDISM
	2449	UNSPECIFIED ACQUIRED HYPOTHYROIDISM
	2409	GOITER, UNSPECIFIED
	2411	NONTOXIC MULTINODULAR GOITER
	2419	UNSPECIFIED NONTOXIC NODULAR GOITER
	2429	THYROTOXICOSIS WITHOUT MENTION OF GOITER OR OTHER CAUSE
	2440	POSTSURGICAL HYPOTHYROIDISM
	2443	OTHER IATROGENIC HYPOTHYROIDISM
	2448	OTHER SPECIFIED ACQUIRED HYPOTHYROIDISM
	2450	ACUTE THYROIDITIS
	2451	SUBACUTE THYROIDITIS
	2452	CHRONIC LYMPHOCYTIC THYROIDITIS
	2461	DYSHORMONOGENIC GOITER
	2468	OTHER SPECIFIED DISORDERS OF THYROID
	24200	TOXIC DIFFUSE GOITER WITHOUT MENTION OF THYROTOXIC CRISIS
	24240	THYROTOXICOSIS FROM ECTOPIC THYROID NODULE,WITHOUT CRISIS
	24290	THYROTOXICOSIS WITHOUT GOITER; HYPERTHYROIDISM
DIABETES	25001	JUVENILE TYPE DIABETES MELLITUS WITHOUT MENTION OF COMPLICATION
	25001	TYPE I(JUVENILE TYPE), DIABETES MELLITUS WITHOUT COMPLICATION,NOT STATED AS UNCONTROLLED
	25003	DIABETES MELLITUS WITHOUT COMPLICATION, TYPE I, UNCONTROLLED
	25011	JUVENILE TYPE DIABETES MELLITUS WITH KETOACIDOSIS
	25011	TYPE I (JUVENILE TYPE), DIABETES MELLITUS WITH KETOACIDOSIS, NOT STATED AS UNCONTROLLED
	25013	DIABETES WITH KETOACIDOSIS, TYPE I, UNCONTROLLED
	25031	JUVENILE DIABETES, NOT STATED AS UNCONTROLLED, WITH OTHER COMA
	25041	TYPE I (JUVENILE TYPE) DIABETES WITH RENAL MANIFESTATIONS NOT STATED AS UNCONTROLLED
	25081	JUVENILE DIABETES, NOT STATED AS UNCONTROLLED,+SPEC. MANIFESTATIN
	25081	TYPE I(JUVENILE TYPE),DIABETES WITH SPECIFIED MANIFESTATION NOT STATED AS UNCONTROLLED
	25083	JUVENILE DIABETES, UNCONTROLLED, + SPEC. MANIFESTATIONS
	2500	DIABETES MELLITUS WITHOUT MENTION OF COMPLICATION
	2508	DIABETES WITH OTHER SPECIFIED MANIFESTATIONS
	24900	SECONDARY DIABETES MELLITUS WITHOUT MENTION OF COMPLICATION, NOT STATED AS UNCONTROLLED, OR UNSPECIFIED
	24901	SECONDARY DIABETES MELLITUS WITHOUT MENT. OF COMPLI. UNCONTROLLED
	25000	TYPE II/UNSPECIFIED TYPE, DIABETES MELLITUS WITHOUT COMPLICATION, NOT STATED AS UNCONTROLLED
	25002	TYPE II OR UNSPECIFIED TYPE, DIABETES MELLITUS, UNCONTROLLED
	25010	TYPE II OR UNSPECIFIED TYPE, DIABETES MELLITUS WITH KETOACIDOSIS, NOT STATED AS UNCONTROLLED
	25050	ADULT-ONSET DIABETES,NOT STATED AS UNCONTROLLED,+OPHTALMIC MANIFE
	25050	TYPE II OR UNSPECIFIED TYPE,DIABETES WITH OPHTALMIC MANIFESTATION NOT STATED AS UNCONTROLLED
	25080	TYPE II OR UNSPECIFIED TYPE,DIABETES WITH SPECIFIED MANIFESTATION NOT STATED AS UNCONTROLLED

**HYPOGLYCEMIA**	2510	HYPOGLYCEMIC COMA (NON-DIABETIC INSULIN COMA)
	2511	OTHER SPECIFIED HYPOGLYCEMIA
	2512	HYPOGLYCEMIA, UNSPECIFIED
**OBESITY**	2779	UNSPECIFIED DISORDER OF METABOLISM
	2780	OVERWEIGHT AND OBESITY
	2781	LOCALIZED ADIPOSITY
	27800	OBESITY, UNSPECIFIED
	27801	MORBID OBESITY
	27802	OVERWEIGHT
**PARATHYROID DISORDERS**	252	DISORDERS OF PARATHYROID GLAND
	2519	UNSPECIFIED DISORDER OF PANCREATIC INTERNAL SECRETION
	2520	HYPERPARATHYROIDISM
	2521	HYPOPARATHYROIDISM
	2532	PANHYPOPITUITARISM
	2533	PITUITARY DWARFISM
	2535	DIABETES INSIPIDUS
	2536	OTHER DISORDERS OF NEUROHYPOPHYSIS
	2540	PERSISTENT HYPERPLASIA OF THYMUS
	2548	OTHER SPECIFIED DISEASES OF THYMUS GLAND
	25200	HYPERPARATHYROIDISM, UNSPECIFIED
	25202	SECONDARY HYPERPARATHYROIDISM, NON-RENAL
**ADRENAL DISEASE**	2550	CUSHING'S SYNDROME
	2551	HYPERALDOSTERONISM
	2552	ADRENOGENITAL DISORDERS
	2553	OTHER CORTICOADRENAL OVERACTIVITY
	2554	CORTICOADRENAL INSUFFICIENCY
	2555	OTHER ADRENAL HYPOFUNCTION
	2558	OTHER SPECIFIED DISORDERS OF ADRENAL GLANDS
	2559	UNSPECIFIED DISORDER OF ADRENAL GLANDS
	25510	HYPERALDOSTERONISM, UNSPECIFIED
	25513	BARTTER'S SYNDROME
	25541	GLUCOCORTICOID DEFICIENCY
	25542	MINERALOCORTICOID DEFICIENCY
**SEX HORMONE DISORDERS**	2561	OTHER OVARIAN HYPERFUNCTION
	2562	POSTABLATIVE OVARIAN FAILURE
	2564	POLYCYSTIC OVARIES
	2572	OTHER TESTICULAR HYPOFUNCTION
	2590	DELAY IN SEXUAL DEVELOPMENT/PUBERTY,NOT ELSEWHERE CLASSIFIED
	2591	PRECOCIOUS SEXUAL DEVELOPMENT AND PUBERTY, NOT ELSEW.CLASSIF.
	25639	OTHER OVARIAN FAILURE
	25951	ANDROGEN INSENSITIVITY SYNDROME
